# Ultra-processed food consumption and body mass index from adolescence to early adulthood: multi-trajectory analysis using data from the 1993 Pelotas (Brazil) birth cohort

**DOI:** 10.1590/0102-311XEN185225

**Published:** 2026-07-31

**Authors:** Michele Krüger Vaz Moreira, Adriana Kramer Fiala Machado, Ana Maria Baptista Menezes, Helen Gonçalves, Fernando C. Wehrmeister

**Affiliations:** 1 Universidade Federal do Rio Grande, Pelotas, Brasil.; 2 Universidade Federal de Pelotas, Pelotas, Brasil.

**Keywords:** Processed Food, Body Mass Index, Adolescent, Risk Behavior, Birth Cohort, Alimentos Processados, Índice de Massa Corporal, Adolescente, Comportamento de Risco, Coorte de Nascimentos, Alimentos Procesados, Índice de Masa Corporal, Adolescente, Conducta de Riesgo, Cohorte de Nacimiento

## Abstract

Given the evidence linking ultra-processed food consumption to adverse health outcomes and the lack of longitudinal studies jointly addressing ultra-processed food consumption and body mass index (BMI), this study identified multi-trajectory groups from adolescence to early adulthood and described their demographic and behavioral characteristics. Data from the 1993 Pelotas (Brazil) birth cohort at ages 15, 18, and 22 (n = 2,890) were analyzed. Sex-stratified multi-trajectory groups of daily ultra-processed food consumption and BMI (z-scores for age and sex) were estimated using Group-Based Trajectory Modelling. Daily ultra-processed food consumption was defined as the number of ultra-processed food items consumed at least once per day. Sixteen NOVA-classified foods were grouped into six categories. Groups were described according to ultra-processed food types, poverty level, physical activity, screen time, smoking, alcohol use, and risk behaviors. Three multi-trajectory groups were identified: two with low ultra-processed food (group 1 and group 2) and one with increasing ultra-processed food intake (group 3). In group 1 (38.7% males; 34% females), BMI increased over time, while in group 2 (39.4% males; 44.5% females) it remained stable. In group 3 (21.9% males; 21.5% females), ultra-processed food consumption increased over time, particularly fast food, soft drinks, and processed meats, while BMI z-scores remained close to zero in males and increased in females. Risk behaviors increased over time in group 3, whereas poverty was less prevalent in group 1. Individuals in group 3 generally exhibited higher ultra-processed food intake and a greater prevalence of risk behaviors across follow-ups, despite BMI remaining within the normal range. Monitoring ultra-processed food consumption may help identify at-risk groups before excess weight becomes evident during adolescence and early adulthood.

## Abstract (pt)

Diante das evidências que associam o consumo de alimentos ultraprocessados a desfechos adversos à saúde e da escassez de estudos longitudinais avaliando conjuntamente o consumo de ultraprocessados e índice de massa corporal (IMC), este estudo identificou grupos de multitrajetórias da adolescência ao início da vida adulta e descreveu suas características demográficas e comportamentais. Foram analisados dados da coorte de nascimentos de 1993 de Pelotas (Brasil) aos 15, 18 e 22 anos (n = 2.890). Grupos de multitrajetórias do consumo diário de ultraprocessados e do IMC (escore-z para idade e sexo), estratificados por sexo, foram estimados utilizando *Group-Based Trajectory Modelling*. O consumo diário de ultraprocessados foi definido pelo número de alimentos consumidos pelo menos uma vez ao dia. Dezesseis alimentos classificados segundo a NOVA foram agrupados em seis categorias. Os grupos foram descritos segundo tipos de ultraprocessados, pobreza, atividade física, tempo de tela, tabagismo, consumo de álcool e comportamentos de risco. Foram identificados três grupos: dois com baixo consumo de ultraprocessados (grupo 1 e grupo 2) e um com aumento do consumo (grupo 3). No grupo 1 (38,7% homens; 34% mulheres), o IMC aumentou, enquanto no grupo 2 (39,4% homens; 44,5% mulheres) permaneceu estável. No grupo 3 (21,9% homens; 21,5% mulheres), o consumo de ultraprocessados aumentou, principalmente fast food, refrigerantes e carnes processadas, enquanto os escores-z de IMC permaneceram próximos de zero nos homens e aumentaram nas mulheres. Comportamentos de risco aumentaram no grupo 3, enquanto a pobreza foi menos prevalente no grupo 1. Grupo 3 apresentou maior consumo de ultraprocessados e mais comportamentos de risco, apesar do IMC normal. O monitoramento do consumo de ultraprocessados pode auxiliar na identificação de grupos em risco anteriormente ao surgimento do excesso de peso.

## Abstract (es)

Dada la evidencia que vincula el consumo de alimentos ultraprocesados con desenlaces adversos para la salud y la escasez de estudios longitudinales sobre consumo de ultraprocesados e índice de masa corporal (IMC), este estudio identificó grupos de multitrayectorias desde la adolescencia hasta la adultez y describió sus características demográficas y conductuales. Se analizaron datos de la cohorte de nacimientos de 1993 de Pelotas (Brasil) a los 15, 18 y 22 años (n = 2.890). Se estimaron grupos de multitrayectorias del consumo diario de ultraprocesados e IMC (puntajes z para edad y sexo), estratificados por sexo, mediante *Group-Based Trajectory Modelling*. El consumo diario de ultraprocesados se definió por el número de alimentos consumidos al menos una vez al día. Dieciséis alimentos clasificados según NOVA fueron agrupados en seis categorías. Los grupos fueron descritos según tipos de ultraprocesados, pobreza, actividad física, tiempo de pantalla, tabaquismo, alcohol y conductas de riesgo. Se identificaron tres grupos: dos con bajo consumo (grupo 1 y grupo 2) y uno con aumento del consumo (grupo 3). En el grupo 1 (38,7% hombres; 34% mujeres), el IMC aumentó; grupo 2 (39,4% hombres; 44,5% mujeres) permaneció estable. En el grupo 3 (21,9% hombres; 21,5% mujeres), aumentó el consumo de ultraprocesados, especialmente comida rápida, refrescos y carnes procesadas; los puntajes z de IMC permanecieron cercanos a cero en hombres y aumentaron en mujeres. Las conductas de riesgo aumentaron en el grupo 3, y la pobreza fue menos prevalente en el grupo 1. El grupo 3 presentó mayor consumo de ultraprocesados y más conductas de riesgo, pese a un IMC normal. El monitoreo del consumo de ultraprocesados puede ayudar a identificar grupos en riesgo antes del exceso de peso.

## Introduction

According to the NOVA classification, ultra-processed foods are industrial formulations made mostly or entirely from substances derived from foods, and are typically ready-to-eat products with low nutritional value ^1^. Soft drinks, salt crackers, and candies are common examples. Ultra-processed food consumption has been increasing worldwide ^2^, with the highest prevalence among children and adolescents ^3^. In the United States, among individuals aged 2-19 years, total energy intake from ultra-processed foods increased from 61.4% in 1999 to 67% in 2018 ^4^. In Brazil, ultra-processed food consumption increased by 4.6 percentage points (p.p.; p < 0.05) between 2002 and 2009, whereas consumption of fresh or minimally processed foods decreased by 1.6p.p. (p < 0.05) during the same period ^5^. Furthermore, Brazil shows a trend toward a high-consumption pattern. Between 2008 and 2018, analyses across different sociodemographic strata revealed that groups with the lowest consumption levels in 2017-2018 experienced the largest increases over the period ^6^.

Systematic reviews and meta-analyses have reported an association between ultra-processed food consumption and a higher risk of chronic diseases, such as type 2 diabetes ^7^, cardiovascular diseases ^8^, depression, and metabolic syndrome ^9^, as well as a higher risk of all-cause mortality ^8^. Additionally, higher ultra-processed food consumption has been associated with an increased risk of overweight and obesity ^10^ ^,^ ^11^ ^,^ ^12^, with more consistent findings among adults than among adolescents ^13^ ^,^ ^14^. In this context, longitudinal studies may help identify distinct patterns of ultra-processed food consumption and weight changes over the life course and contribute to a better understanding of this relationship.

Overweight during adolescence is considered a public health concern due to its increasing global incidence. By 2035, it is expected that 20% of adolescent boys and 18% of adolescent girls will experience obesity ^15^. Adolescence is a critical period of biological development, characterized by high energy requirements, making it a crucial window to develop healthy habits that may persist into adulthood ^16^. Evidence suggests that weight changes during this stage differ according to sex. A study of Chinese children and adolescents showed that in the last 25 years (1985-2010), obesity prevalence increased in both sexes, especially among boys ^17^. Analyses exploring sex differences can help elucidate disparities in weight changes and their long-term consequences ^18^ ^,^ ^19^ ^,^ ^20^.

International data show an increase in both ultra-processed food consumption and obesity prevalence ^21^ ^,^ ^22^. However, the relationship between weight gain and ultra-processed food consumption requires further investigation, especially during early life stages ^13^. Multi-trajectory analysis is an approach that identifies distinct groups of individuals who follow similar patterns in relation to more than one characteristic ^23^. To the best of our knowledge, no longitudinal studies have jointly evaluated body mass index (BMI) and ultra-processed food consumption in either adults or adolescents. This approach may help clarify how these two outcomes interact across the life course and whether sex differences are present. This study aimed to identify multi-trajectory groups of ultra-processed food consumption and BMI from adolescence to early adulthood and to describe these trajectories according to types of ultra-processed foods consumed, as well as demographic and behavioral characteristics.

## Methods

### Study population and design

This study used longitudinal data from the 1993 Pelotas (Brazil) birth cohort. In 1993, all maternity hospitals in the city of Pelotas, Rio Grande do Sul State, were visited daily to identify and recruit mothers and their newborns living in urban areas of the municipality. Of the 5,265 mothers identified, 5,249 agreed to participate in the perinatal study. Follow-ups with all the sample were conducted when participants were 11, 15, 18, and 22 years old. At each follow-up visit, sociodemographic, socioeconomic, behavioral, and other characteristics were collected. Further information is available elsewhere ^24^ ^,^ ^25^. Follow-up rates at ages 15, 18, and 22 were 85.7% (n = 4,349; deaths = 147), 81.4% (n = 4,106; deaths = 164), and 76.3% (n = 3,810; deaths = 193), respectively. This study used data from the 15-, 18-, and 22-year follow-up waves and included participants with available BMI and dietary intake data.

### Outcomes

#### Ultra-processed food

Ultra-processed food consumption was obtained using *Food Frequency Questionnaires* (FFQ) administered at ages 15, 18, and 22 years. At 15 years, the FFQ assessed consumption frequency (1-10 times) and whether intake occurred daily, weekly, monthly, or annually. At 18 and 22 years, the tool evaluated both frequency and quantity consumed. Frequency of consumption was categorized as: never or less than once per month; once to thrice per month; once per week; two to four times per week; five to six times per week; once per day; two to four times per day; and five or more times per day. Further information is available elsewhere ^26^ ^,^ ^27^ ^,^ ^28^. Data from the 11-year follow-up were not considered, as the FFQ addresses a limited number of ultra-processed foods. Sixteen ultra- processed foods items (hot dog/hamburger, pizza, mayonnaise, soda, sweetened fruit juice, sandwich biscuits, ice cream, candy, chocolate powder, chocolate, salty crackers, chips, sausages, ham/mortadella, yogurt, and cheese) were assessed at all three follow-ups and classified according to the NOVA classification ^1^ and were included in the analysis. For each ultra-processed food item, consumption frequencies of at least once per day were classified as daily consumption. The number of ultra-processed food item consumed daily was then modeled using the multi-trajectory approach.

To describe trajectory groups, ultra-processed foods items were categorized into the following groups: fast food (hot dog/hamburger, pizza, and mayonnaise); soft drinks (soda and sweetened fruit juice); candies (sweet biscuits, ice cream, candy, chocolate powder, and chocolate); snacks (salty crackers and chips); processed meats (sausages and ham/mortadella); and dairy products (yogurt and cheese). This grouping strategy increased the interpretability of ultra-processed food consumption profiles and is consistent with approaches used to describe dietary patterns in epidemiological studies ^6^ ^,^ ^29^ ^,^ ^30^ ^,^ ^31^.

#### Body mass index

At all follow-up visits, anthropometric measurements were performed by trained individuals. At age 15, height was measured using an aluminum stadiometer with 0.1cm precision, and weight was measured using an electronic scale (Tanita, https://tanita.com/) with 0.1kg precision. At ages 18 and 22, height was measured using a stadiometer with 0.1cm precision, and weight was assessed using an electronic scale integrated with air-displacement plethysmography equipment (BodPod Gold Standard. COSMED; https://www.cosmed.com/en/). BMI was calculated as weight (kg) divided by height squared (m²), ensuring comparability across the three follow-ups.

BMI was classified based on the World Health Organization (WHO) growth curve, which standardizes BMI values according to sex and age. A BMI z-score between -1 and +1 standard deviations (SD) was considered within the normal range. At 22 years, the BMI-for-age z-score was derived from the 19-year reference, as the WHO BMI curve plateaus in late adolescence and becomes less age-dependent. At this stage, the +1 SD and +2 SD thresholds approximate the classification of overweight and obesity, respectively ^32^.

### Demographic, behavioral, and socioeconomic characteristics

Trajectory groups were defined according to demographic, behavioral, and socioeconomic variables. Socioeconomic status was assessed using an asset index derived from principal component analysis, based on information on household appliances ownership, characteristics of the residence, sanitation, and existence of assets. This index was divided into quintiles, with the first representing the poorest and the fifth the wealthiest. The first two quintiles were considered as low socioeconomic status ^33^.

Physical activity was assessed using a list of leisure and commuting activities at ages 15 and 22 ^34^ and on the *International Physical Activity Questionnair*e (IPAQ) at age 18 ^35^. Although the IPAQ may overestimate physical activity levels ^36^, both instruments measured weekly minutes of moderate-to-vigorous physical activity, enabling temporal comparability. Physical activity (minutes per week) was categorized into terciles to reflect its relative distribution across follow-ups, with the first tercile indicating the lowest physical activity level. Screen time was assessed using questions about time spent (hours per day) watching television and using video games or computers. The highest tercile was classified as high screen time. Trajectory groups were also described according to alcohol use (“yes” for consumption in the past month), smoking (“yes” for smoking in the past week), and accumulation of risk behaviors, which was defined as the presence of at least two of the following: lowest level of physical activity, high screen time, alcohol consumption in the past month, and smoking in the past week. 

### Statistical analyses

Group-based multi-trajectory modeling was used to identify joint trajectory groups of ultra-processed foods consumption and BMI across follow-ups. This approach extends Group-Based Trajectory Modeling (GBTM), a form of finite mixture modeling for longitudinal data that identifies a specified number of groups of individuals who follow similar trajectories over time ^37^. Multi-trajectory modeling enables the simultaneous assessment of interrelationships between two or more outcomes over time. In this approach, the resulting groups represent empirical longitudinal patterns rather than predefined classifications. Individuals are assigned to the group for which they have the highest posterior probability of membership.

Initially, sex-specific single-trajectory models were fitted for each outcome using *traj* in Stata 18.0 (https://www.stata.com). Ultra-processed food consumption was modeled using a zero-inflated Poisson distribution, while BMI was modeled using a censored normal distribution. The number of trajectories was selected based on the Bayesian information criterion (BIC), Akaike information criterion (AIC), group average posterior probability (higher than 70%), odds of correct classification (OCC higher than 5%), minimum group size (higher than 5%), statistical significance, and interpretability of the obtained trajectories. Several combinations were tested, including up to quadratic terms, to determine the best fit for each outcome. Subsequently, combinations of the selected single-outcome models were tested to estimate the multi-trajectory model. The final model was selected according to the same goodness-of-fit criteria previously described ^23^. Medians were calculated based on the number of the 16 individual ultra-processed food items consumed daily. Interquartile ranges and the Kruskal-Wallis test were used to describe ultra-processed food consumption, while standard deviations and analysis of variance (ANOVA) were used to describe BMI across multi-trajectory groups, stratified by sex and follow-up wave. Multi-trajectory groups were described according to ultra-processed foods categories, with corresponding 95% confidence intervals (95%CI). Ultra-processed food consumption between ages 15 and 22 was assessed and classified as increased, decreased, or stable based on 95%CI overlap. Poverty (based on the wealth index), lowest physical activity level, alcohol use, smoking, screen time, and accumulation of risk behaviors were also described according to trajectory group, stratified by sex. Data from the 22-year follow-up were collected using REDCap software (https://redcapbrasil.com.br/) ^38^. All analyses were performed using Stata version 18.0.

### Ethics approval

The study protocols were approved by the Research Ethics Committee of the Federal University of Pelotas. All participants provided written informed consent. For those under 18 years of age, consent was obtained from their parents or legal guardians. The 15-, 18-, and 22-year follow-ups were approved under protocol numbers 158/2007, 05/11, and 1.250.366, respectively. 

## Results

The multi-trajectory analysis included 2,890 individuals with complete data at ages 15, 18, and 22. A total of 2,359 individuals were excluded due to missing BMI or ultra-processed food consumption information, or due to loss to follow-up. Compared with the original cohort, the analytical sample included a lower proportion of individuals whose mothers had lower educational attainment (Table 1).

**Table 1:** Perinatal characteristics of participants included and not included in the multi-trajectory analyses. 1993 Pelotas (Brazil) birth cohort.

Characteristics	Included (n = 2,890)		Not included (n = 2,359)	
	%	95%CI	%	95%CI
Sex				
Male	48.2	46.4; 50.1	51.3	49.3; 53.3
Female	51.8	49.9; 53.6	48.7	46.7; 50.7
Family income (terciles)				
Q1 (poorest)	42.3	40.5; 44.1	44.6	42.6; 46.7
Q2	29.6	28.0; 31.3	26.3	24.5; 28.1
Q3	28.1	26.5; 29.8	29.1	27.3; 31.0
Maternal schooling (years)				
0-4	26.2	24.6; 27.8	30.2	28.4; 32.1
5-8	48.3	46.5; 50.1	43.7	41.7; 45.7
≥ 9	25.5	23.9; 27.1	26.1	24.4; 27.9
Low birth weight	9.2	8.2; 10.3	10.5	9.3; 11.8
Maternal obesity	5.0	4.3; 5.9	4.5	3.7; 5.5
Smoking during pregnancy	31.8	30.2; 33.6	35.3	33.4; 37.2
Alcohol drinking during pregnancy	5.3	4.5; 6.1	4.9	4.1; 5.8

95%CI: 95% confidence interval.

**Figure 1:**
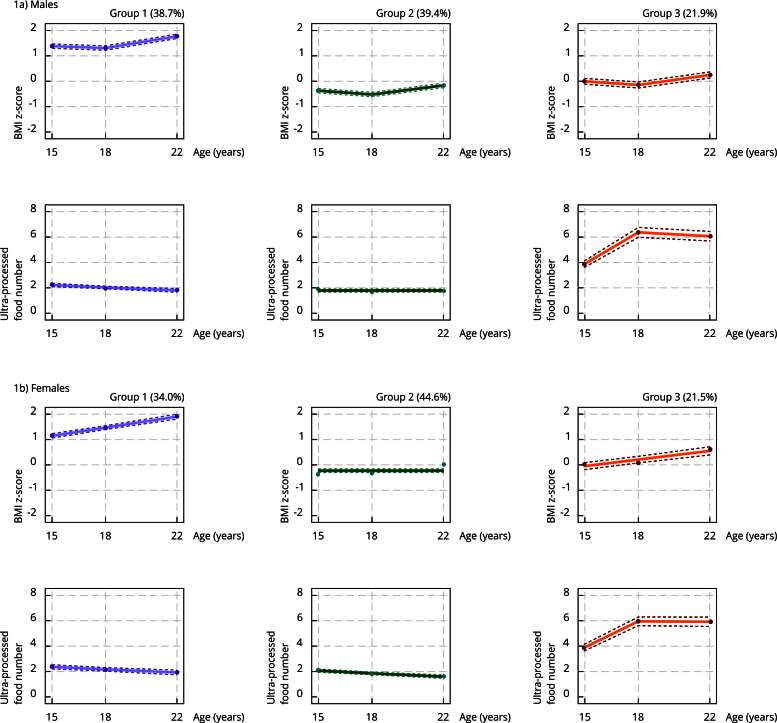
Fitted three-group multi-trajectories of BMI and daily ultra-processed food consumption among males and females at ages 15, 18, and 22. 1993 Pelotas (Brazil) birth cohort.

Note: daily ultra-processed food consumption was defined as frequencies equal to or greater than once per day. Males − group 1: initially high and moderately increasing BMI, with low and decreasing ultra-processed food consumption; group 2: average and moderately increasing BMI, with consistently low ultra-processed food consumption; group 3: average and moderately increasing BMI, with increasing and persistently high ultra-processed food consumption. Females − group 1: high and steadily increasing BMI, with low and decreasing ultra-processed food consumption; group 2: stable average BMI, with low and decreasing ultra-processed food consumption; group 3: average and steadily increasing BMI, with increasing and persistently high ultra-processed food consumption.

The three-group model was selected for each outcome and for males and females. The multi-trajectory models included combinations of linear and quadratic terms, based on parameters and interpretability of the trajectory groups. The model specifications and parameter estimates are shown in Supplementary Material (https://cadernos.ensp.fiocruz.br/static//arquivo/supl-e00185225_9955.pdf). Figure 1a presents the multi-trajectory model for males. Group 1 (38.7%) had initially high BMI values, which increased modestly from ages 15 to 22 years, while ultra-processed food consumption was low and progressively decreased over time. Group 2 (39.4%) had BMI-for-age z-scores close to zero, with a slight increase, and consistently low ultra-processed food consumption across all ages. Group 3 (21.9%) showed moderate increases in BMI over time but showed the steepest rise in ultra-processed food consumption, peaking at ages 18 and 22. Among females (Figure 1b), three trajectory groups were also identified, although with different patterns. Group 1 (34%) showed high BMI values with a steady increase, accompanied by initially low and decreasing ultra-processed food consumption. Group 2 (44.6%) had average and stable BMI values and declining ultra-processed food consumption. Group 3 (21.5%) showed a moderate increase in BMI and a marked increase in ultra-processed food consumption, peaking at age 18 and remaining high at age 22. These groups represent the predominant longitudinal patterns observed in the sample rather than all possible individual-level trajectories. Individuals whose observed patterns did not closely match a group were assigned based on the highest posterior probability of membership (minimum 0.70).

Table 2 presents the median number of ultra-processed food items consumed daily and the mean BMI z-score for each group, stratified by sex and follow-up wave. Groups 1 and 2 showed low ultra-processed food consumption across follow-ups, whereas group 3 showed the highest consumption at all ages. Differences between trajectory groups were consistent at each follow-up in both sexes (p < 0.001). BMI z-scores also varied between trajectory groups across all ages (p < 0.001), with group 1 presenting the highest mean values (1.31 to 1.79 for males and 1.32 to 2.11 for females) and group 2 the lowest (-0.54 to -0.19 for males and -0.38 to -0.01 for females). These differences were consistently observed at ages 15, 18, and 22. Table 3 describes the prevalence of sociodemographic and behavioral characteristics according to multi-trajectory groups. Among males, the lowest prevalence of poverty was observed in group 1 at ages 15 (30.1; 95%CI: 26.4; 34.1) and 18 (28.6%; 95%CI: 24.9; 32.5), whereas the highest prevalence was observed in group 3 at age 15 (47.3%; 95%CI: 41.7; 53.0). In groups 1 and 2, the prevalence of the lowest physical activity level, alcohol consumption, smoking, and risk behavior increased over time. In group 3, alcohol consumption increased from 47.3% to 81.1%, smoking from 11.6% to 41.7%, and risk behaviors from 33.3% to 63.3%. At age 22, group 3 had a higher prevalence of smoking (41.7%; 95%CI: 36.2; 47.3) and high screen time (40.3%; 95%CI: 34.9; 46.0) compared to other groups. Among females, the lowest prevalence of poverty was observed in group 2 across all ages. In this group, alcohol consumption increased from 61.6% to 73%, while the lowest physical activity level decreased from 49.6% to 38.6%. In group 3, increases were observed in alcohol use from 56% to 71.3%, smoking from 19.7% to 31.9%, high screen time from 22.3% to 28%, and two or more risk behaviors from 43.9% to 52.3%.

**Table 2:** Description of trajectory groups according to ultra-processed food consumption and body mass index (BMI) z-score by age and sex. 1993 Pelotas (Brazil) birth cohort.

	Group 1		Group 2		Group 3		
Males						
Ultra-porcessed foods number	Median	IQR	Median	IQR	Median	IQR	p-value *
Follow-up							
15 years	2.0	1.0-3.0	2.0	1.0-3.0	4.0	2.0-5.0	< 0.001
18 years	1.0	0.0-3.0	1.0	0.0-2.0	6.0	4.0-8.5	< 0.001
22 years	1.0	0.0-3.0	1.0	0.0-3.0	6.0	4.0-8.0	< 0.001
BMI z-score/age	Mean	95%CI	Mean	95%CI	Mean	95%CI	p-value **
Follow-up							
15 years	1.38	1.32; 1.45	-0.38	-0.45; -0.32	0.00	-0.11; 0.10	< 0.001
18 years	1.31	1.24; 1.38	-0.54	-0.60; -0.48	-0.15	-0.25; -0.05	< 0.001
22 years	1.79	1.72; 1.86	-0.19	-0.26; -0.12	0.25	0.14; 0.40	< 0.001
Females						
Ultra-processed food number	Median	IQR	Median	IQR	Median	IQR	p-value *
Follow-up							
15 years	2.0	1.0-3.0	2.0	1.0-3.0	4.0	2.0-6.0	< 0.001
18 years	2.0	1.0-3.0	1.0	1.0-3.0	6.0	4.0-8.0	< 0.001
22 years	1.0	3.0-3.0	1.0	0.0-2.0	6.0	4.0-6.0	< 0.001
BMI z-score/age	Mean	95%CI	Mean	95%CI	Mean	95%CI	p-value **
Follow-up							
15 years	1.32	1.25; 1.39	-0.38	-0.44; -0.32	0.01	-0.80; 0.11	< 0.001
18 years	1.60	1.55; 1.70	-0.35	-0.41; -0.29	0.08	-0.17; 0.18	< 0.001
22 years	2.11	2.04; 2.19	-0.01	-0.07; 0.06	0.61	0.50; 0.72	< 0.001

95%CI: 95% confidence interval; IQR: interquartile range; SD: standard deviation.

Note: ultra-processed number: 16 ultra-processed food items (hot dog/hamburger, pizza, mayonnaise, soda, sweetened fruit juice, sweets biscuits, ice cream, candy, chocolate powder, chocolate, salty crackers, chips, sausages, ham/mortadella, yogurt, cheese);

Males - group 1: initially high and moderately increasing BMI, with low and decreasing ultra-processed food consumption; group 2: average and moderately increasing BMI, with consistently low ultra-processed food consumption; group 3: average and moderately increasing BMI, with increasing and persistently high ultra-processed food consumption.

Females - group 1: high and steadily increasing BMI, with low and decreasing ultra-processed food consumption; group 2: stable average BMI, with low and decreasing ultra-processed food consumption; group 3: average and steadily increasing BMI and increasing, with persistently high ultra-processed food consumption.

* Kruskal-Wallis test;

** ANOVA.

**Table 3:** Prevalence of poverty and risk behaviors according to the multi-trajectory group, age, and sex. 1993 Pelotas (Brazil) birth cohort.

	Group 1		Group 2		Group 3	
	%	95%CI	%	95%CI	%	95%CI
Males						
Poorest group (age)						
15 years	30.1	26.4; 34.1	40.1	36.1; 44.3	47.3	41.7; 53.0
18 years	28.6	24.9; 32.5	41.6	37.6; 45.8	41.5	36.0; 47.1
22 years	30.4	26.7; 34.4	38.5	34.6; 43.3	37.7	32.4; 43.3
Physical inactivity (age)						
15 years	20.6	17.4; 24.2	21.8	18.6; 25.5	17.0	13.2; 21.7
18 years	19.5	16.3; 23.0	22.5	19.2; 26.2	17.1	13.2; 21.8
22 years	28.3	24.7; 32.2	30.7	27.0; 34.7	21.0	16.8; 26.0
Alcohol use (age)						
15 years	54.7	50.5; 59.0	52.0	47.8; 56.2	47.3	41.6; 53.0
18 years	76.3	72.6; 79.7	74.4	70.5; 77.8	76.6	71.4; 81.0
22 years	78.7	74.9; 82.0	77.8	74.1; 81.2	81.1	76.1; 85.2
Smoking (age)						
15 years	10.1	7.8; 13.0	11.9	9.5; 15.0	11.6	8.4; 15.8
18 years	20.5	17.3; 24.1	18.4	15.3; 21.8	30.8	25.8; 36.2
22 years	30.9	27.1; 34.9	23.6	20.3; 27.4	41.7	36.2; 47.3
High screen time (age)						
15 years	37.5	33.5; 41.7	35.5	31.6; 39.5	33.3	28.2; 38.9
18 years	30.9	27.1; 34.9	26.7	23.2; 30.6	31.1	26.1; 36.6
22 years	30.3	26.6; 34.3	30.7	27.0; 34.7	40.3	34.9; 46.0
Risk behavior (age)						
15 years	36.2	32.3; 40.3	35.3	31.4; 39.4	33.3	28.2; 38.9
18 years	47.5	43.3; 51.7	44.7	40.6; 48.9	50.5	44.8; 56.1
22 years	53.9	49.7; 58.0	51.3	47.1; 55.4	63.3	57.7; 68.6
Females						
Poorest group (age)						
15 years	43.4	39.1; 47.7	33.5	30.0; 37.1	50.3	44.7; 55.9
18 years	46.0	41.7; 50.3	34.5	31.0; 38.1	54.8	49.1; 60.3
22 years	48.8	44.5; 53.1	36.7	33.1; 40.4	52.1	46.5; 57.7
Physical inactivity (age)						
15 years	44.9	40.7; 49.3	49.6	45.9; 53.4	41.3	35.9; 46.9
18 years	43.1	38.9; 47.4	47.6	43.9; 51.4	45.4	39.9; 51.0
22 years	39.5	35.4; 43.8	38.6	35.0; 42.3	41.1	35.7; 46.7
Alcohol use (age)						
15 years	65.2	60.9; 69.2	61.6	57.8; 65.2	56.0	50.3; 61.5
18 years	72.3	68.3; 76.0	70.0	66.4; 73.3	71.1	65.8; 76.0
22 years	72.3	68.2; 76.1	73.0	69.4; 76.3	71.3	65.7; 76.3
Smoking (age)						
15 years	20.0	16.7; 23.7	19.0	16.2; 22.1	19.7	15.6; 24.5
18 years	22.4	19.0; 26.2	17.0	14.4; 20.0	25.2	20.7; 30.4
22 years	24.3	20.8; 28.2	18.9	16.2; 22.1	31.9	26.9; 37.4
High screen time (age)						
15 years	27.8	24.1; 31.9	26.4	23.3; 29.9	22.3	18.0; 27.3
18 years	25.7	22.1; 29.7	24.6	21.5; 27.9	27.5	22.8; 32.8
22 years	25.7	22.1; 29.6	25.0	21.9; 28.4	28.0	23.2; 33.3
Risk behavior (age)						
15 years	51.6	47.2; 55.9	50.2	46.5; 54.0	43.9	38.5; 49.6
18 years	56.7	52.4; 61.0	51.8	48.0; 55.5	59.7	54.1; 65.0
22 years	51.9	47.6; 56.2	46.7	43.0; 50.5	52.3	46.7; 57.9

95%CI: 95% confidence interval. BMI: body mass index.

Note: risk behavior: at least two behaviors (lowest physical activity level; alcohol use; smoking, and high screen time), Males - group 1: initially high and moderately increasing BMI, with low and decreasing ultra-processed food consumption; group 2: average and moderately increasing BMI, with consistently low ultra-processed food consumption; group 3: average and moderately increasing BMI, with increasing and persistently high ultra-processed food consumption. Females - group 1: high and steadily increasing BMI, with low and decreasing ultra-processed food consumption; group 2: stable average BMI, with low and decreasing ultra-processed food consumption; group 3: average and steadily increasing BMI, with increasing and persistently high ultra-processed food consumption.

**Figure 2:**
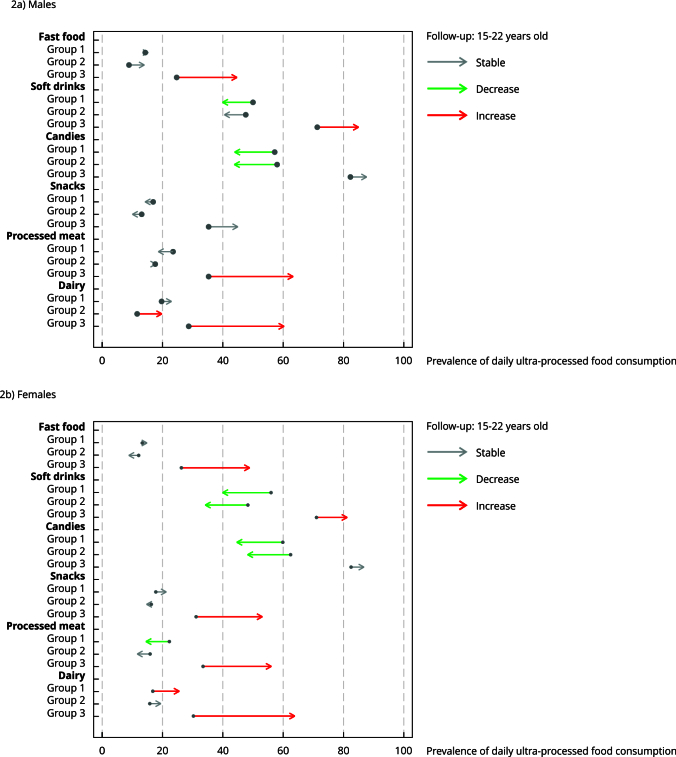
Changes in the prevalence of daily consumption of ultra-processed food groups from 15 to 22 years of age, according to multi-trajectory groups among males and females. 1993 Pelotas (Brazil) birth cohort.

Note: daily ultra-processed food consumption was defined as frequencies equal to or greater than once per day. Males - group 1: initially high and moderately increasing body mass index (BMI), with low and decreasing ultra-processed food consumption; group 2: average and moderately increasing BMI, with consistently low ultra-processed food consumption; group 3: average and moderately increasing BMI, with increasing and persistently high ultra-processed food consumption. Females - group 1: high and steadily increasing BMI, with low and decreasing ultra-processed food consumption; group 2: stable average BMI, with low and decreasing ultra-processed food consumption; group 3: average and steadily increasing BMI, with increasing and consistently high ultra-processed food consumption. “Increase” and “decrease” indicate non-overlapping 95% confidence interval; otherwise, trends were considered stable.

Figure 2 shows changes in the prevalence of daily consumption of ultra-processed food categories by sex and multi-trajectory group from ages 15-22. For both sexes, group 3 consistently presented the highest prevalence across all ultra-processed food groups. Fast-food consumption increased over time in group 3 for both males and females, whereas groups 1 and 2 remained relatively stable. Soft drink consumption decreased in groups 1 and 2 in both sexes, whereas group 3 showed an increase. Snack consumption increased only in group 3 among females and remained relatively stable in the other groups. The prevalence of processed meat consumption increased in group 3 in both sexes, and decreased among females in group 1. Dairy consumption increased in groups 2 and 3 for both males and females. Candy consumption decreased in groups 1 and 2 in both sexes, remained stable in group 3 among males, and decreased among females in group 3.

## Discussion

This longitudinal study investigated the joint trajectories of BMI and ultra-processed food consumption from adolescence to early adulthood. To the best of our knowledge, this is the first study to evaluate simultaneous changes in these outcomes using a multi-trajectory approach. Three trajectory groups were identified for both sexes. Within the GBTM framework, these trajectories represent empirical longitudinal patterns rather than predefined classifications, and individuals are assigned to the group for which they have the highest posterior probability of membership. BMI and ultra-processed food consumption differed across trajectory groups in both sexes. Group 3 was characterized by high ultra-processed food consumption and more risk behaviors, despite maintaining BMI within the normal range. This cohort experienced adolescence during a period of rapid expansion in ultra-processed food availability in Brazil and was assessed between 2008 and 2015. Current adolescents may face even greater exposure, which could lead to different ultra-processed food and BMI trajectories.

Ultra-processed food consumption and excess weight are public health issues that can impair quality of life and lead to negative long-term outcomes ^8^ ^,^ ^39^. Despite consistent evidence of harm, ultra-processed foods are becoming increasingly prevalent in diets worldwide ^6^ ^,^ ^40^ ^,^ ^41^. The consumption of these products also places a substantial indirect burden on public healthcare systems, as it is associated with several noncommunicable diseases ^8^ ^,^ ^12^ ^,^ ^42^. Strategies to reduce the purchase and consumption of ultra-processed foods require a comprehensive set of policies, including fiscal measures, regulation over production and distribution, and initiatives that encourage trade and consumption of fresh and minimally processed foods ^22^. Identifying trajectory groups with high ultra-processed food intake before excess weight manifests may provide a window of opportunity for targeted public health interventions.

The results of multi-trajectory analysis among males suggest that BMI changes related to ultra-processed food consumption may occur later in adolescence or early adulthood. Biological mechanisms related to pubertal growth and body composition changes ^43^ may temporarily mask excess weight gain attributable to ultra-processed food consumption, making short-term associations between ultra-processed food intake and BMI more difficult to detect during this life stage ^12^. Moreover, consistent intake over time, even if low, may adversely affect nutritional status in later life stages ^44^. Females in group 3 showed consumption trajectories similar to those observed among males in the same group but presented adequate BMI at age 15, followed by an increasing BMI across the three waves. Sexual maturation usually occurs earlier in females than in males ^43^. At age 15, girls may experience a slower linear growth rate than boys, which may increase susceptibility to excess weight gain during this stage, making the impact of consumption more immediate. Additionally, adolescents with overweight − especially girls, who tend to be more concerned about body image ^45^ − may underreport food consumption and underestimate their intake of these products ^46^ ^,^ ^47^. The relationship between ultra-processed food consumption and weight gain over time, especially during adolescence, warrants further investigation ^14^ ^,^ ^44^.

Among males, the highest poverty prevalence was observed in group 3 at age 15, while among females, the lowest prevalence was observed in group 2 across all follow-ups. Evidence on the relationship between ultra-processed food consumption and socioeconomic parameters remains inconsistent. In high-income countries, ultra-processed food consumption tends to be inversely associated with socioeconomic position, while in low-and middle-income countries, this association is generally direct ^2^ ^,^ ^48^ ^,^ ^49^. Although the greatest consumption of these products is still observed in high-income countries, the rate of consumption growth has been more pronounced in low- and middle-income countries, requiring greater attention ^2^ ^,^ ^22^.

Our study observed that group 3 showed a greater increase in the prevalence of two or more risk behaviors over time in both sexes. The co-occurrence of risk factors tends to intensify from adolescence to early adulthood ^50^ ^,^ ^51^ ^,^ ^52^, highlighting the need for actions to preserve the health of this population and mitigate long-term negative consequences. In both sexes, group 3 presented the highest prevalence of ultra-processed food consumption, with no reduction in any food group over time. The trajectories observed in this group are consistent with global evidence demonstrating a steady rise in ultra-processed food consumption ^1^ ^,^ ^2^ ^,^ ^53^. The increasing prevalence of obesity and noncommunicable diseases underscores the importance of monitoring dietary patterns in this population ^2^. Contemporary adolescents may be exposed to these products earlier and more intensely than previous generations. In Brazil, data evaluating ultra-processed food consumption among individuals aged ≥ 10 years between 2008 and 2018 showed an average increase of 5.5% ^6^. Further studies should investigate emerging consumption patterns in contexts of expanding food availability. The unbalanced composition of ultra-processed food (high energy density and poor nutritional value), combined with their high palatability and marketing strategies that emphasize convenience, may contribute to high consumption, particularly among adolescents, who are more susceptible to less conscious dietary choices ^22^ ^,^ ^54^ ^,^ ^55^.

Among the strengths of this study is its longitudinal design, based on data from a large and representative birth cohort in Southern Brazil. The use of multi-trajectory models enabled the simultaneous assessment of ultra-processed food consumption and BMI, providing a more comprehensive understanding of the relationship between adolescence and early adulthood. Additionally, sex-stratified analysis revealed significant differences in the observed patterns.

However, some limitations must be considered. First, follow-up losses were greater among individuals with lower maternal educational attainment level. These differences may lead to an underestimation of ultra-processed food consumption and BMI level, as both outcomes tend to be higher in poorer strata ^56^ ^,^ ^57^ ^,^ ^58^. Different FFQs were used over time, and it was not possible to explore quantities consumed, as this information was not available in all follow-ups. To minimize information bias, the analysis was restricted to ultra-processed food items consistently assessed at all time points. The consumption was self-reported and may have been subject to recall bias, which is inherent to this type of instrument. Cheese and yogurt were categorized as ultra-processed foods in this paper, although depending on the ingredients used, they could be considered processed foods; however, label evaluation was not feasible due to limitations in the FFQ. Despite this, a large amount of the cheese available in markets is pre-packaged or processed and can be classified as ultra-processed food ^59^ ^,^ ^60^. GBTM assumes distinct, mutually exclusive latent groups, potentially oversimplifying continuous heterogeneity, and group assignment is probabilistic rather than causal. Nonetheless, the approach is useful for identifying longitudinal patterns that are difficult to detect with traditional methods ^37^. Another limitation is the reliance solely on BMI, as standardized data on other body composition measures were unavailable across all analyzed follow-ups. However, BMI is a widely used measure with internationally established criteria and shows a strong correlation with fat mass index, especially among young individuals ^61^. Future analyses should aim to incorporate additional body composition measures. BMI-for-age z-scores were used to maximize accuracy. To ensure comparability across follow-ups, z-scores at age 22 were calculated using 19-year reference values, given the plateauing of the WHO growth curve in late adolescence and the approximate correspondence between +1/+2 SD and the adult thresholds for overweight and obesity (25/30kg/m^2^), which makes BMI less age-dependent and minimizes potential misclassification. 

Our findings indicate that ultra-processed food consumption may help identify important risk groups during adolescence and early adulthood, even when excess weight is not yet evident. This highlights the importance of going beyond BMI alone when monitoring adolescent health, as dietary patterns can anticipate future health risks that are not yet reflected in body weight.

## Conclusions

This study outlined multi-trajectory groups of BMI and ultra-processed food consumption from adolescence to early adulthood, highlighting sex differences. Despite presenting a normal BMI, some groups of boys and girls showed increasing ultra-processed food consumption and presented more risk behaviors. Ultra-processed food intake among adolescents and young adults may signal particularly vulnerable groups who engage in other unhealthy practices. Further prospective studies involving multiple life stages are needed to expand knowledge of the relationship between ultra-processed food and BMI.

## Data Availability

The research data are available upon request to the corresponding author.

## References

[1] Monteiro CA, Cannon G, Levy RB, J-C Moubarac, Louzada ML, Rauber F (2019). Ultra-processed foods: what they are and how to identify them.. Public Health Nutr.

[2] Baker P, Machado P, Santos T, Sievert K, Backholer K, Hadjikakou M (2020). Ultra-processed foods and the nutrition transition global, regional and national trends, food systems transformations and political economy drivers. Obes Rev.

[3] Marino M, Puppo F, Del Bo' C, Vinelli V, Riso P, Porrini M (2021). A systematic review of worldwide consumption of ultra-processed foods: findings and criticisms.. Nutrients.

[4] Wang L, Martínez Steele E, Du M, Pomeranz JL, O'Connor LE, Herrick KA (2021). Trends in consumption of ultraprocessed foods among US youths aged 2-19 years, 1999-2018.. JAMA.

[5] Martins APB, Levy RB, Claro RM, Moubarac JC, Monteiro CA (2013). Participação crescente de produtos ultraprocessados na dieta brasileira (1987-2009). Rev Saúde Pública.

[6] Louzada MLC, Cruz GL, Silva KAAN, Grassi AGF, Andrade GC, Rauber F (2023). Consumption of ultra-processed foods in Brazil distribution and temporal evolution 2008-2018. Rev Saúde Pública.

[7] Chen Z, Khandpur N, Desjardins C, Wang L, Monteiro CA, Rossato SL (2023). Ultra-processed food consumption and risk of type 2 diabetes three large prospective U.S. cohort studies. Diabetes Care.

[8] Pagliai G, Dinu M, Madarena MP, Bonaccio M, Iacoviello L, Sofi F (2021). Consumption of ultra-processed foods and health status a systematic review and meta-analysis. Br J Nutr.

[9] Lane MM, Lotfaliany M, Hodge AM, O'Neil A, Travica N, Jacka FN (2023). High ultra-processed food consumption is associated with elevated psychological distress as an indicator of depression in adults from the Melbourne Collaborative Cohort Study.. J Affect Disord.

[10] Askari M, Heshmati J, Shahinfar H, Tripathi N, Daneshzad E (2005). Ultra-processed food and the risk of overweight and obesity a systematic review and meta-analysis of observational studies. Int J Obes.

[11] Costa CS, Del-Ponte B, Assunção MCF, Santos IS (2018). Consumption of ultra-processed foods and body fat during childhood and adolescence a systematic review. Public Health Nutr.

[12] Lane MM, Davis JA, Beattie S, Gómez-Donoso C, Loughman A, O'Neil A (2021). Ultraprocessed food and chronic noncommunicable diseases: a systematic review and meta-analysis of 43 observational studies.. Obes Rev.

[13] Dicken SJ, Batterham RL (2024). Ultra-processed food and obesity what is the evidence?. Curr Nutr Rep.

[14] Louzada MLC, Costa CS, Souza TN, Cruz GL, Levy RB, Monteiro CA (2021). Impacto do consumo de alimentos ultraprocessados na saúde de crianças, adolescentes e adultos revisão de escopo. Cad Saúde Pública.

[15] Lobstein T, Jackson-Leach R, Moodie ML, Hall KD, Gortmaker SL, Swinbum BA (2015). Child and adolescent obesity part of a bigger picture. Lancet.

[16] Mikkilä V, Räsänen L, Raitakari OT, Pietinen P, Viikari J (2004). Longitudinal changes in diet from childhood into adulthood with respect to risk of cardiovascular diseases the Cardiovascular Risk in Young Finns Study. Eur J Clin Nutr.

[17] Song Y, Wang H-J, Dong B, Ma J, Wang Z, Agardh A (2016). 25-year trends in gender disparity for obesity and overweight by using WHO and IOTF definitions among Chinese school-aged children a multiple cross-sectional study. BMJ Open.

[18] Shah B, Tombeau Cost K, Fuller A, Birken CS, Anderson LN (2020). Sex and gender differences in childhood obesity contributing to the research agenda. BMJ Nutr Prev Health.

[19] Bonsergent E, Benie-Bi J, Baumann C, Agrinier N, Tessier S, Thilly N (2012). Effect of gender on the association between weight status and health-related quality of life in adolescents. BMC Public Health.

[20] Solmi F, Sharpe PH, Gage SH, Maddock J, Lewis G, Patalay P (2021). Changes in the prevalence and correlates of weight-control behaviors and weight perception in adolescents in the UK, 1986-2015. JAMA Pediatr.

[21] Beslay M, Srour B, Méjean C, Allès B, Fiolet T, Debras C (2020). Ultra-processed food intake in association with BMI change and risk of overweight and obesity a prospective analysis of the French NutriNet-Santé cohort. PLOS Med.

[22] Monteiro CA, Cannon GJ (2019). The role of the transnational ultra-processed food industry in the pandemic of obesity and its associated diseases problems and solutions. World Nutr.

[23] Nagin DS, Jones BL, Passos VL, Tremblay RE (2018). Group-based multi-trajectory modeling. Stat Methods Med Res.

[24] Gonçalves H, Assunção MC, Wehrmeister FC, Oliveira IO, Barros FC, Victora CG (2014). Cohort profile update the 1993 Pelotas (Brazil) birth cohort follow-up visits in adolescence. Int J Epidemiol.

[25] Gonçalves H, Wehrmeister FC, Assunção MCF, Tovo-Rodrigues L, Oliveira IO, Murray J (2018). Cohort profile update the 1993 Pelotas (Brazil) birth cohort follow-up at 22 years. Int J Epidemiol.

[26] Gigante DP, Reichert FF, Hallal PC, Souza RV, Neutzling MB, Vieira MFA (2010). Dietary assessment in the 1993 Pelotas (Brazil) birth cohort study comparing energy intake with energy expenditure. Cad Saúde Pública.

[27] Schneider BC, Motta JVS, Muniz LC, Bielemann RM, Madruga SW, Orlandi SP (2016). Desenho de um questionário de frequência alimentar digital autoaplicado para avaliar o consumo alimentar de adolescentes e adultos jovens coortes de nascimentos de Pelotas, Rio Grande do Sul. Rev Bras Epidemiol.

[28] Vaz JS, Buffarini R, Kac G, Bielemann RM, Oliveira I, Menezes AB (2018). Dietary patterns are associated with blood lipids at 18-year-olds a cross-sectional analysis nested in the 1993 Pelotas (Brazil) birth cohort. Nutr J.

[29] Islam MR, Rahman SM, Rahman MM, Pervin J, Rahman A, Ekström E-C (2022). Gender and socio-economic stratification of ultra-processed and deep-fried food consumption among rural adolescents A cross-sectional study from Bangladesh. PLOS One.

[30] Lee H, Yim Y, Cho Y, Oh J, Kim S, Son Y (2025). Long-term trends and patterns in ultra-processed food consumption among Korean adults from 1998 to 2022. Sci Rep.

[31] Costa CS, Steele EM, Faria FR, Monteiro CA (2022). Score of ultra-processed food consumption and its association with sociodemographic factors in the Brazilian National Health Survey, 2019. Cad Saúde Pública.

[32] Onis M, Onyango AW, Borghi E, Siyam A, Nishida C, Siekmann J (2007). Development of a WHO growth reference for school-aged children and adolescents. Bull World Health Organ.

[33] Wehrmeister FC, Ferreira LZ, Amouzou A, Blumenberg C, Fayé C, Ricardo LIC (2024). Identifying and characterizing the poorest urban population using National Household Surveys in 38 cities in Sub-Saharan Africa. J Urban Health.

[34] Bastos JP, Araujo CLP, Hallal PC (2008). Prevalence of insufficient physical activity and associated factors in Brazilian adolescents. J Phys Act Health.

[35] Craig CL, Marshall AL, Sjöström M, Bauman AE, Booth ML, Ainsworth BE (2003). International physical activity questionnaire 12-country reliability and validity. Med Sci Sports Exerc.

[36] Lee PH, Macfarlane DJ, Lam TH, Stewart SM (2011). Validity of the International Physical Activity Questionnaire Short Form (IPAQ-SF) a systematic review. Int J Behav Nutr Phys Act.

[37] Nagin DS (2005). Group-based modeling of development.

[38] Harris PA, Taylor R, Thielke R, Payne J, Gonzalez N, Conde JG (2009). Research electronic data capture (REDCap) - a metadata-driven methodology and workflow process for providing translational research informatics support. J Biomed Inform.

[39] World Obesity Day Atlases Obesity atlas 2023..

[40] Martínez Steele E, Baraldi LG, Louzada MLC, Moubarac JC, Mozaffarian D, Monteiro CA (2016). Ultra-processed foods and added sugars in the US diet evidence from a nationally representative cross-sectional study. BMJ Open.

[41] Moubarac JC, Parra DC, Cannon G, Monteiro CA (2014). Food classification systems based on food processing significance and implications for policies and actions: a systematic literature review and assessment. Curr Obes Rep.

[42] Nilson EAF, Andrade RCS, Brito DA, Oliveira ML (2020). Custos atribuíveis a obesidade, hipertensão e diabetes no Sistema Único de Saúde, Brasil, 2018. Rev Panam Salud Pública.

[43] Christie D, Viner R (2005). Adolescent development. BMJ.

[44] De Amicis R, Mambrini SP, Pellizzari M, Foppiani A, Bertoli S, Battezzati A (2022). Ultra-processed foods and obesity and adiposity parameters among children and adolescents a systematic review. Eur J Nutr.

[45] Hill AJ, Draper E, Stack J (1994). A weight on children's minds body shape dissatisfactions at 9-years old. Int J Obes Relat Metab Disord.

[46] Jones L, Ness A, Emmett P (2021). Misreporting of energy intake from food records completed by adolescents associations with sex, body image, nutrient, and food group intake. Front Nutr.

[47] Martínez-Arroyo A, Duarte Batista L, Corvalán Aguilar C, Fisberg RM (2022). Misreporting of energy intake is related to specific food items in low-middle income chilean adolescents. Children.

[48] Schnabel L, Kesse-Guyot E, Allès B, Touvier M, Srour B, Hercberg S (2019). Association between ultraprocessed food consumption and risk of mortality among middle-aged adults in France. JAMA Intern Med.

[49] Simões BS, Barreto SM, Molina MCB, Luft VC, Duncan BB, Schmidt MI (2018). Consumption of ultra-processed foods and socioeconomic position a cross-sectional analysis of the Brazilian Longitudinal Study of Adult Health (ELSA-Brasil). Cad Saúde Pública.

[50] Crespo PA, Nunes BP, Barros FC, Gonçalves H, Menezes AMB, Wehrmeister FC (2022). Multimorbidity and simultaneity of health risk factors, from adolescence to early adulthood 1993 Pelotas Birth Cohort. Prev Med.

[51] Silveira ADS, Santos JEM, Cancela MC, Souza DLB (2023). Estimativa de multimorbidade em jovens brasileiros resultados da Pesquisa Nacional de Saúde 2019. Ciênc Saúde Colet.

[52] van den Akker M, Dieckelmann M, Hussain MA, Bond-Smith D, Muth C, Pati S (2022). Children and adolescents are not small adults: toward a better understanding of multimorbidity in younger populations.. J Clin Epidemiol.

[53] Levy RB, Andrade GC, Cruz GL, Rauber F, Louzada MLC, Claro RM (2022). Três décadas da disponibilidade domiciliar de alimentos segundo a NOVA - Brasil, 1987-2018. Rev Saúde Pública.

[54] Gamboa-Gamboa T, Blanco-Metzler A, Vandevijvere S, Ramirez-Zea M, Kroker-Lobos MF (2019). Nutritional content according to the presence of front of package marketing strategies the case of ultra-processed snack food products purchased in Costa Rica. Nutrients.

[55] Williams DM (2024). Ultra-processed foods and the strategic manipulation of our evolved motivational tendencies. Prev Med Rep.

[56] Kim T, Knesebeck O (2018). Income and obesity what is the direction of the relationship? A systematic review and meta-analysis. BMJ Open.

[57] Inoue K, Seeman TE, Nianogo R, Okubo Y (2023). The effect of poverty on the relationship between household education levels and obesity in U S. children and adolescents: an observational study. Lancet Reg Health Am.

[58] Chavez-Ugalde IY, Vocht F, Jago R, Adams J, Ong KK, Forouhi NG (2024). Ultra-processed food consumption in UK adolescents distribution, trends, and sociodemographic correlates using the National Diet and Nutrition Survey 2008/09 to 2018/19. Eur J Nutr.

[59] Monteiro CA, Cannon G, Moubarac JC, Levy RB, Louzada MLC, Jaime PC (2018). The UN Decade of Nutrition, the NOVA food classification and the trouble with ultra-processing. Public Health Nutr.

[60] Zhong G-C, Gu H-T, Peng Y, Wang K, Wu Y-Q-L, Hu T-Y (2021). Association of ultra-processed food consumption with cardiovascular mortality in the US population: long-term results from a large prospective multicenter study.. Int J Behav Nutr Phys Act.

[61] Jeong S-M, Lee DH, Rezende LFM, Giovannucci EL (2023). Different correlation of body mass index with body fatness and obesity-related biomarker according to age, sex and race-ethnicity.. Sci Rep.

