# Supplementary material for: Ultra-processed food consumption and body mass index from adolescence to early adulthood: multi-trajectory analysis using data from the 1993 Pelotas (Brazil) birth cohort
Source: Cad Saude Publica. 2026 Jul 31;42:e00185225. doi: 10.1590/0102-311XEN185225 (PMC13431993; doi:10.1590/0102-311XEN185225)
Supplement: Supplementary Material [file 1678-4464-csp-42-EN185225-s.pdf]

Supplementary Material

Table S1 Description of parameters (BIC, AIC, Trajectory shapes, APP and estimates) of the multi-trajectory group models of ultra-processed food andbody mass index (BMI), in both sexes. The Pelotas 1993 birth cohort study, Brazil.

**Multi-trajectory models**		**BIC (number of subjects)**	**AIC**	**Trajectory shapes**	**Group**	**APP**	**Estimates**		
							**Parameter**	**Estimate (SE)**	**p-value**
**Male**	**BMI z-score**	-15118.36	-15071.2	2 2 2	G1	92.3	Intercept	7.252 (1.272)	< 0.001
							Linear	-0.696 (0.139)	< 0.001
							Quadratic	0.02 (0.004)	< 0.001
					G2	90.9	Intercept	5.836 (1.263)	< 0.001
							Linear	-0.716 (0.139)	< 0.001
							Quadratic	0.02 (0.004)	< 0.001
					G3	90.2	Intercept	6.4 (1.692)	< 0.001
							Linear	-0.742 (0.185)	< 0.001
							Quadratic	0.021 (0.005)	< 0.001
	**Ultra-processed food number**			1 0 2	G1	92.3	Intercept	1.249 (0.132)	< 0.001
							Linear	-0.03 (0.007)	< 0.001
					G2	90.9	Intercept	0.581 (0.031)	< 0.001
					G3	90.2	Intercept	-8.06 (0.978)	< 0.001
							Linear	1.012 (0.106)	< 0.001
							Quadratic	-0.026 (0.003)	< 0.001
**Female**	**BMI z-score**	-16275.78	-16235.9	1 0 1	G1	91.1	Intercept	-0.407 (0.149)	0.006
							Linear	0.113 (0.008)	< 0.001
					G2	91.1	Intercept	-0.228 (0.037)	< 0.001
					G3	91.9	Intercept	-1.343 (0.190)	< 0.001
							Linear	0.086 (0.010)	< 0.001
	**Ultra-processed food number**			1 1 2	G1	91.1	Intercept	1.315 (0.133)	< 0.001
							Linear	-0.03 (0.008)	< 0.001
					G2	91.1	Intercept	1.285 (0.129)	< 0.001
							Linear	-0.037 (0.007)	< 0.001
					G3	91.9	Intercept	-6.366 (0.968)	< 0.001
							Linear	0.824 (0.105)	< 0.001
							Quadratic	-0.021 (0.003)	< 0.001

BIC: Bayesian information criterion; AIC: Akaike information criterion; APP: Group Average Posterior Probability; SE: standard error.

Note: trajectory shape indicates the polynomial order of time (0 = intercept only; 1 = linear; 2 = quadratic).
